# Supplementary material for: Content and Stability of Hydroxycinnamic Acids during the Production of French Fries Obtained from Potatoes of Varieties with Light-Yellow, Red and Purple Flesh
Source: Antioxidants (Basel). 2023 Jan 29;12(2):311. doi: 10.3390/antiox12020311 (PMC9951911; doi:10.3390/antiox12020311)
Supplement: Supplementary file 1 [file antioxidants-12-00311-s001.zip › antioxidants-2163262-supplementary.pdf]

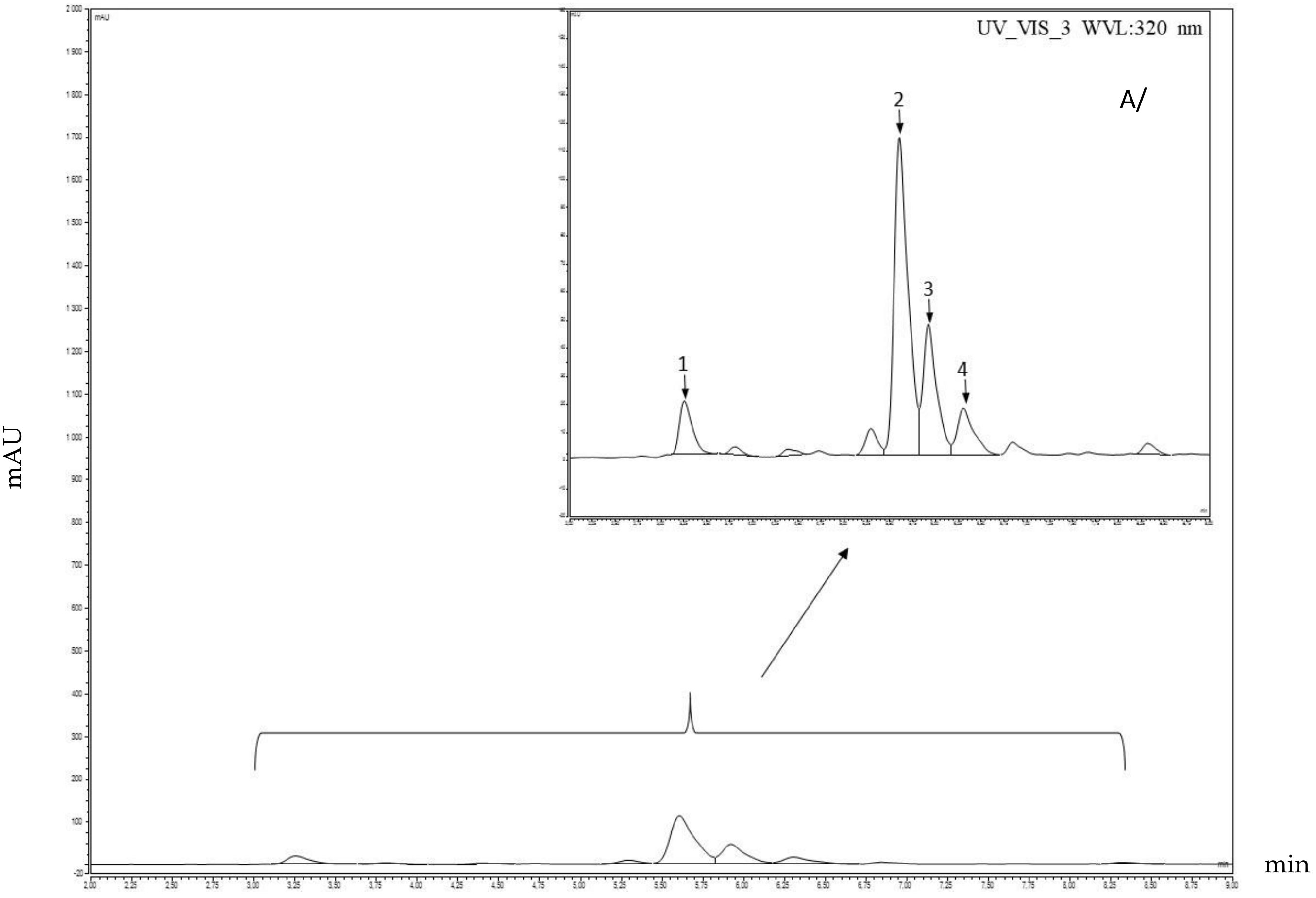

Figure S1. A

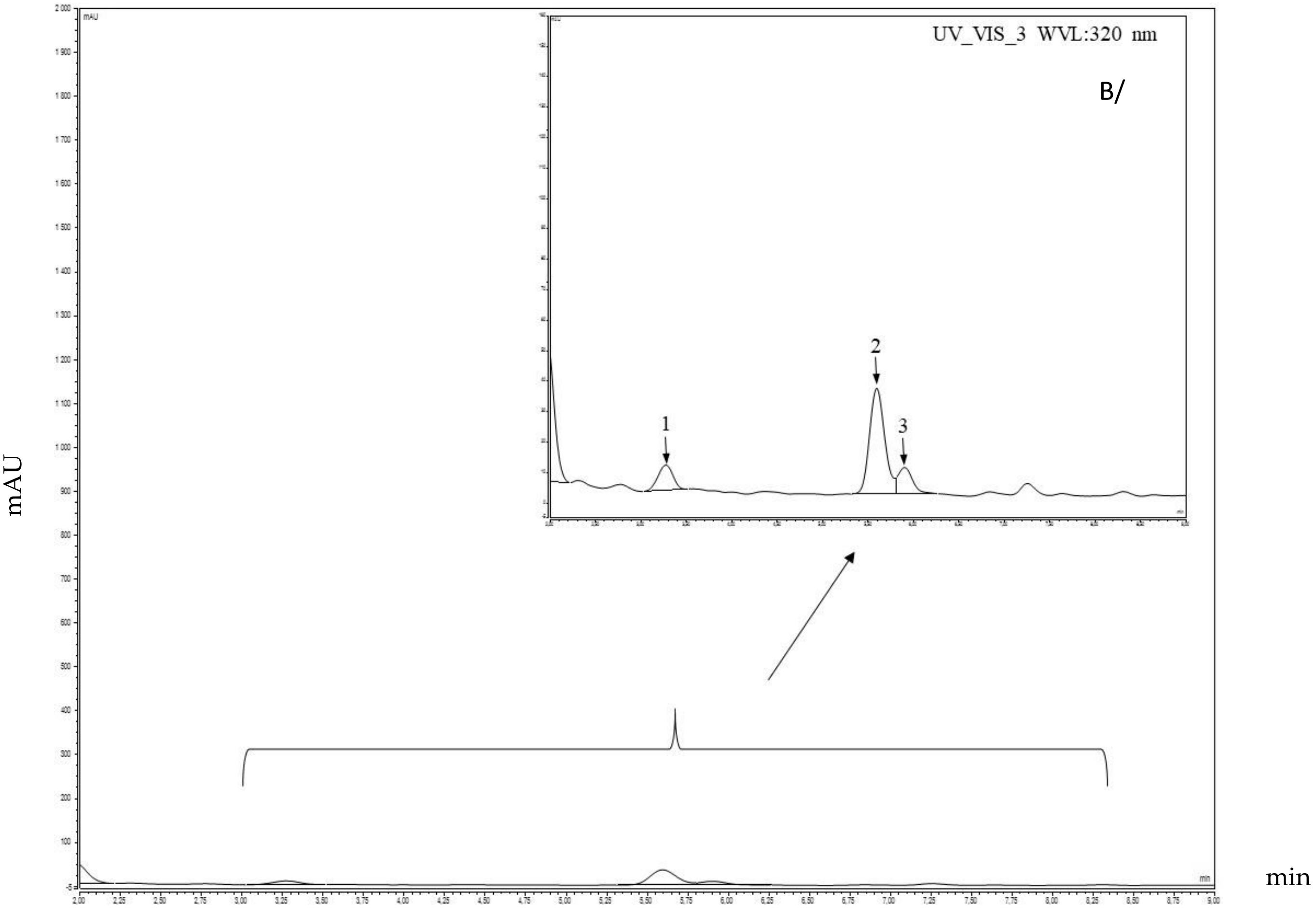

Figure S1. B (Cont.)

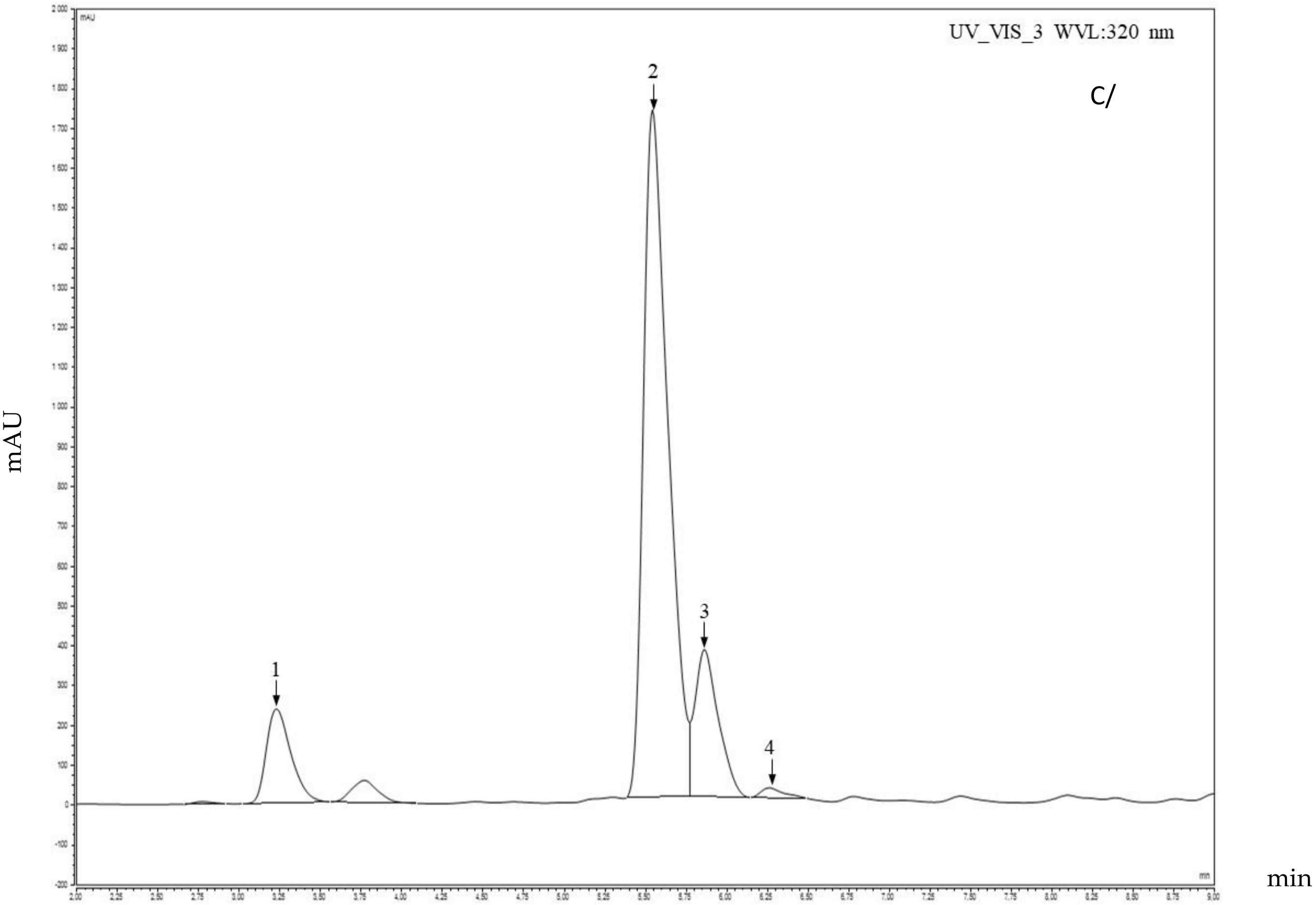

Figure S1. C (Cont.)

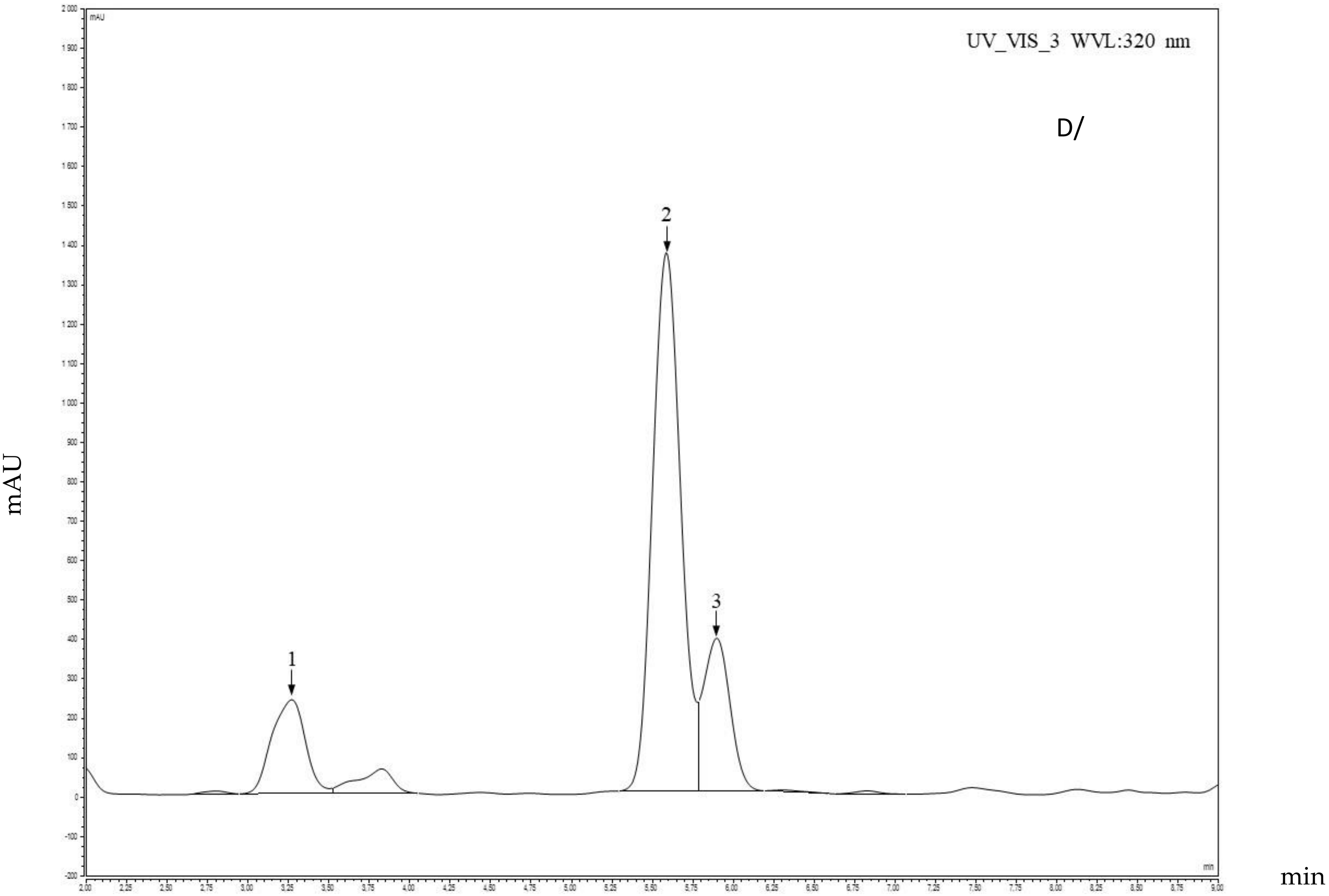

Figure S1. D (Cont.)

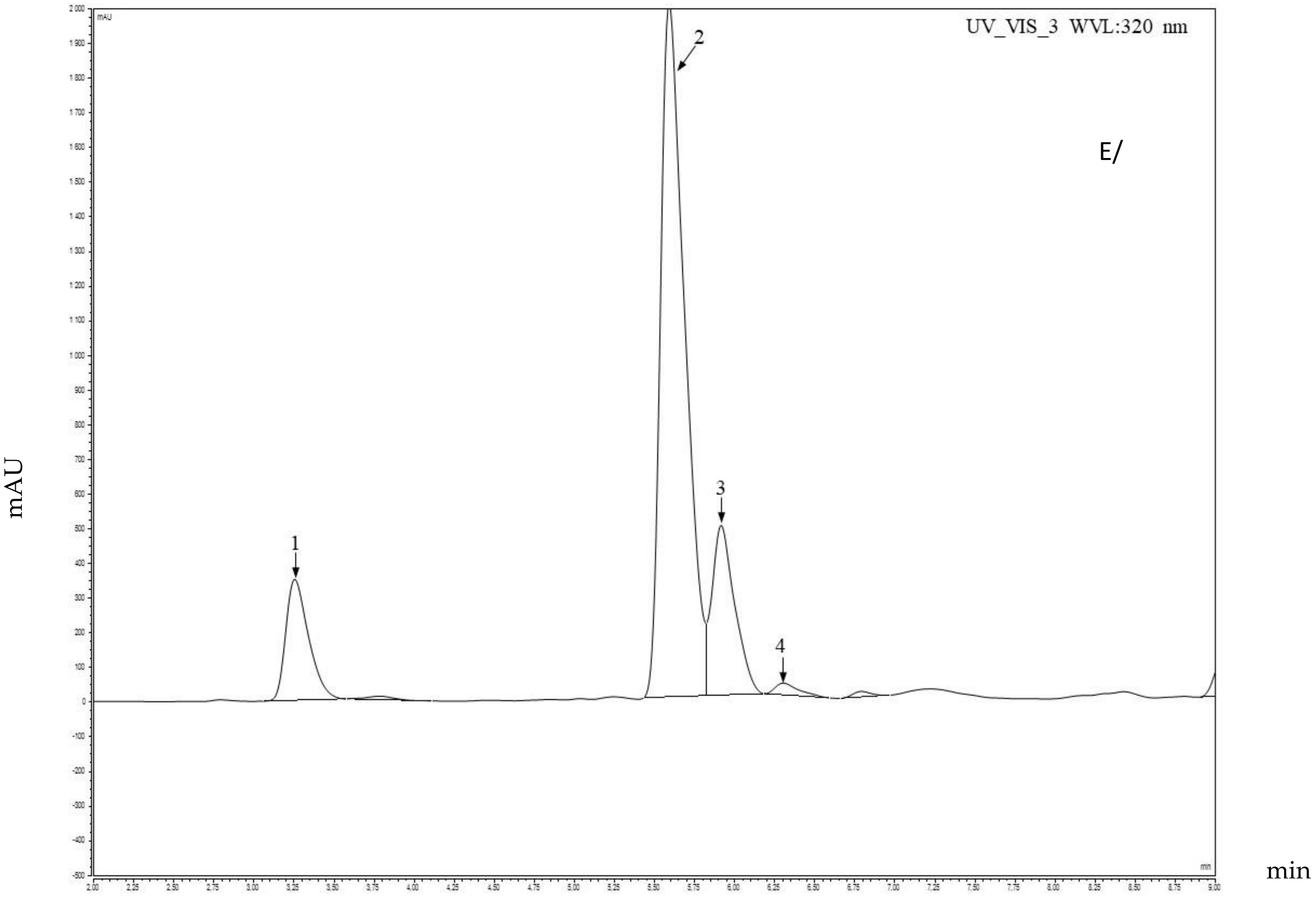

Figure S1. E (Cont.)

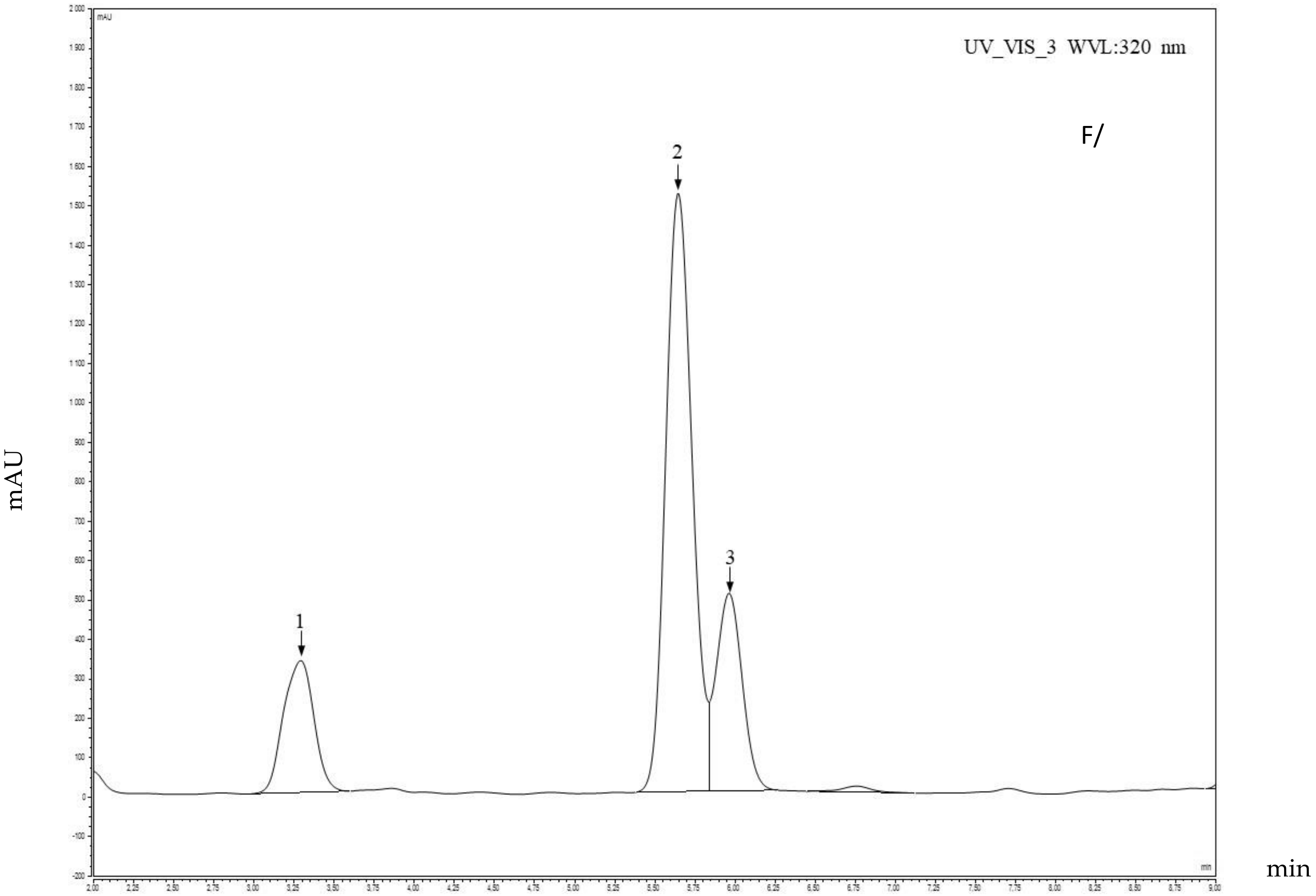

Figure S1 A-F. HPLC-PDA chromatograms (320nm) of hydroxycinnamic acids: **A/** - hydroxycinnamic acids in bright-fleshed potatoes of Lady Anna (LA) variety; **B/** - hydroxycinnamic acids in French fries obtained from bright-fleshed potatoes of Lady Anna (LA) variety; **C/** - hydroxycinnamic acids in red-fleshed potatoes of Mulberry Beauty (MB) variety; **D/** - hydroxycinnamic acids in French fries obtained from red-fleshed potatoes of Mulberry Beauty (MB) variety; **E/** - hydroxycinnamic acids in blue-fleshed potatoes of Violet Queen (VQ) variety; **F/** - hydroxycinnamic acids in French fries obtained from blue-fleshed potatoes of Violet Queen (VQ) variety; Peak 1 - neochlorogenic acid (3-CQA); Peak 2 - chlorogenic acid (5-CQA); Peak 3 - cryptochlorogenic acid (4-CQA); Peak 4 - caffeic acid (CA)

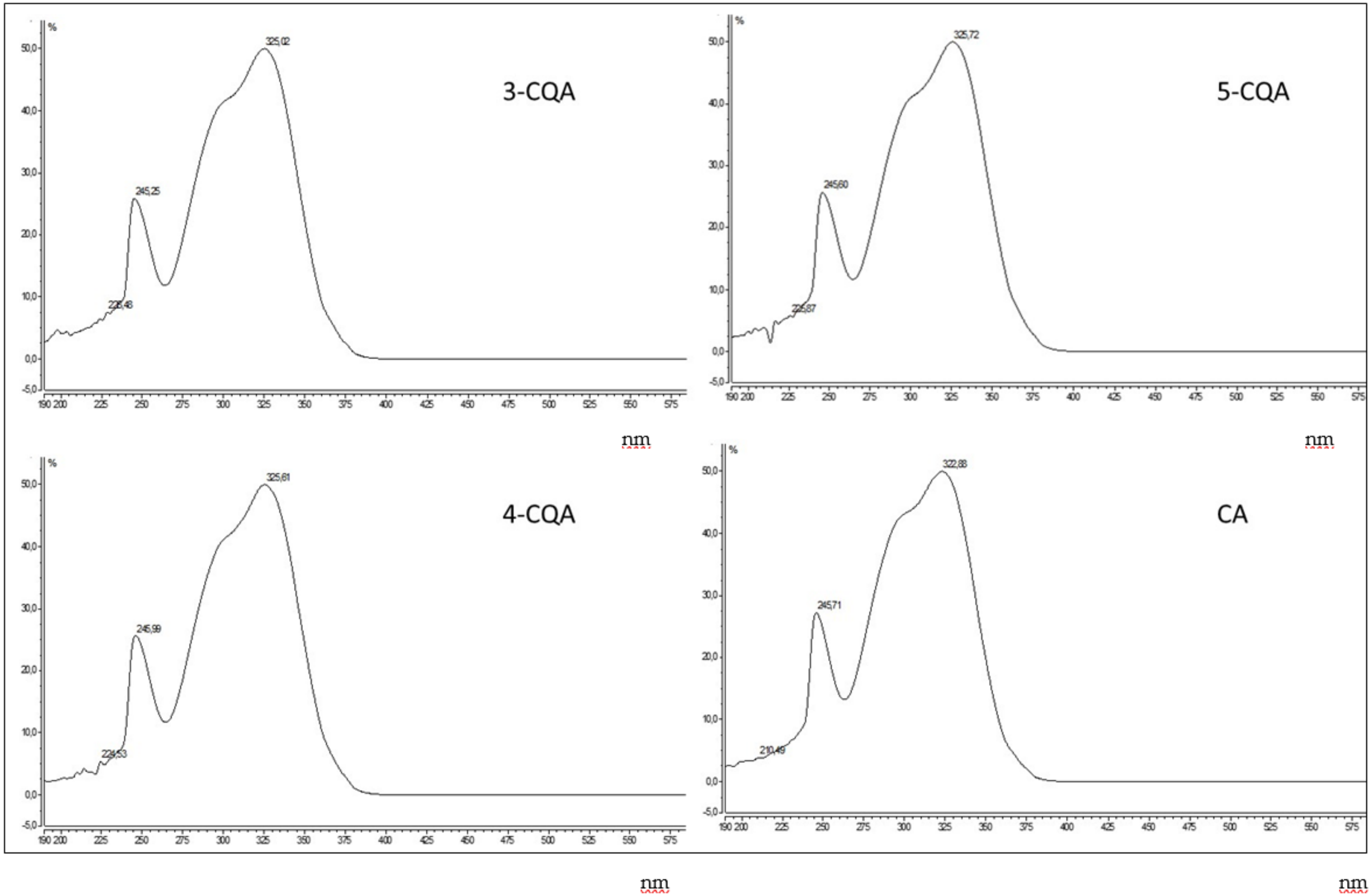

Figure S2. UV-Vis spectrum of chlorogenic acid (5-CQA; 5-O-caffeoylquinic acid), cryptochlorogenic acid (4-CQA; 4-O-caffeoylquinic acid), neochlorogenic acid (3-CQA; 3-O-caffeoylquinic acid) and caffeic acid (CA; 3,4-dihydroxycinnamic acid) standards

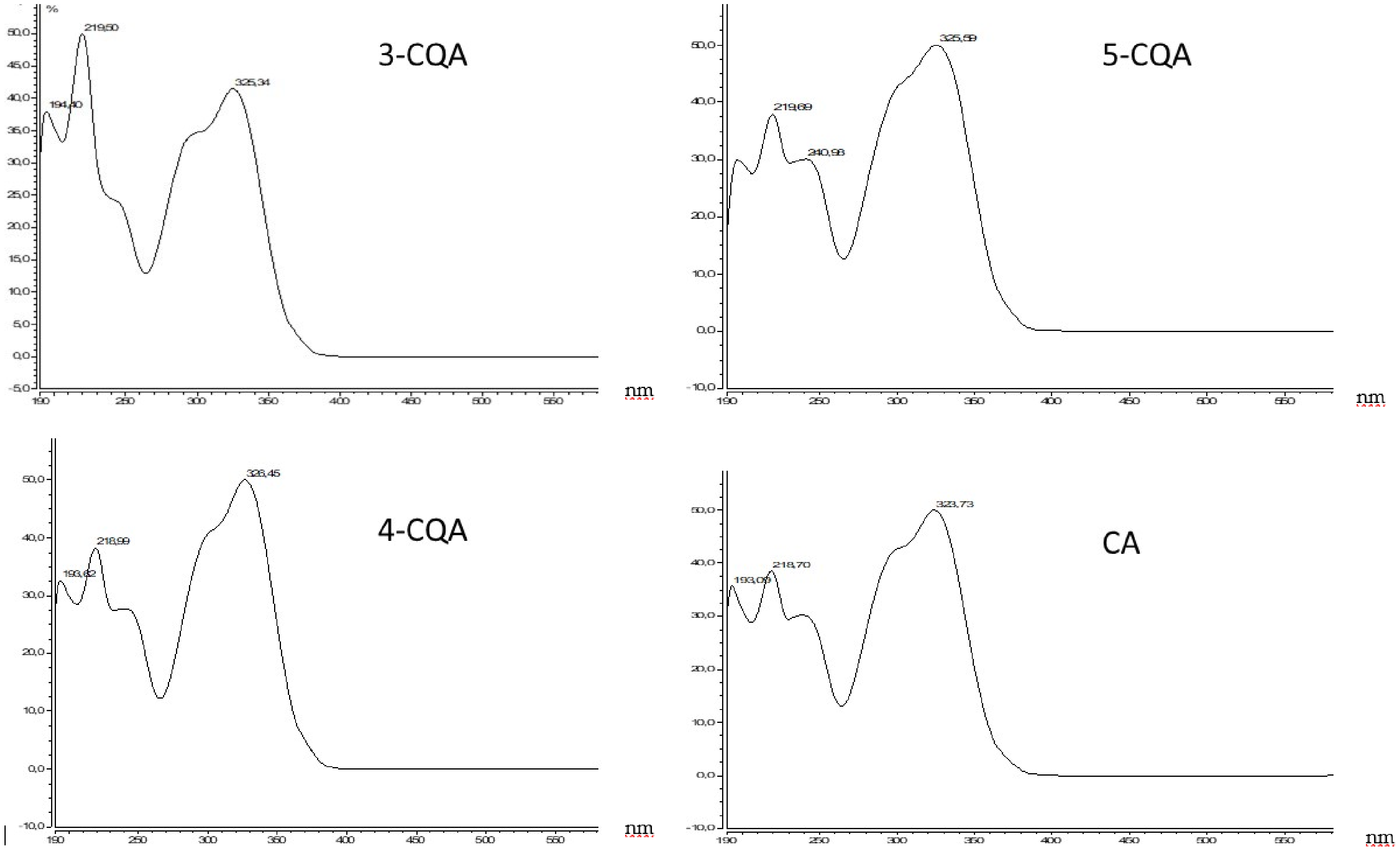

Figure S3. UV-Vis spectrum of chlorogenic acid (5-CQA; 5-O-caffeoylquinic acid), cryptochlorogenic acid (4-CQA; 4-O-caffeoylquinic acid), neochlorogenic acid (3-CQA; 3-O-caffeoylquinic acid) and caffeic acid (CA; 3,4-dihydroxycinnamic acid) in an example of investigated potato sample
